# Multifocal Arterial Thrombosis during Thalidomide Therapy: Case Report and Review of the Literature

**DOI:** 10.1155/2009/372073

**Published:** 2009-08-19

**Authors:** Monica Ferri, Gianluca Faggioli, Francesca Fratesi, Andrea Stella

**Affiliations:** Department of Vascular Surgery, University of Bologna, Via Massarenti 9, 40138 Bologna, Italy

## Abstract

*Introduction*. Thalidomide has been associated with both venous and arterial thrombotic events. *Case Presentation*. A 66-years old man during thalidomide therapy for myeloma experienced acute right arm ischemia, emergently treated with thrombectomy and, on postoperative day one left side weakness with right internal carotid thrombosis. *Discussion*. Because of the increased risk of arterial thrombosis complication, prophylactic therapy with ASA or anticoagulation during thalidomide administration is mandatory.

## 1. Introduction

The antiangiogenic properties of thalidomide have brought to its use in the treatment of multiple myeloma (MM) and other neoplastic disease. However, thalidomide has been associated with venous thrombotic events and, more rarely, with arterial thrombisis. 

 We report a case of multiple arterial thrombosis in a MM patient treated with thalidomide.

## 2. Case Presentation

**Figure 1:**
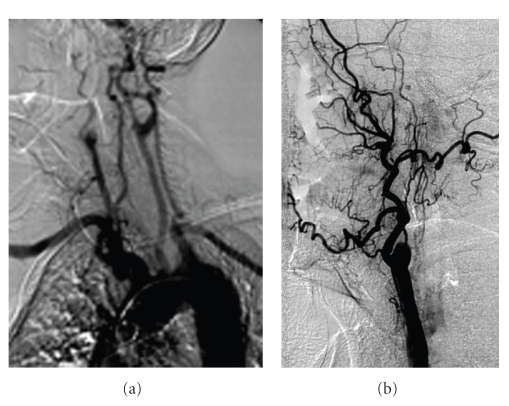
(a) Angiography of the aortic arch. (b) The angiography shows internal carotid occlusion.

A 66-years old man had a one-year history of MM treated with chemotherapy and stem cell transplantation followed by a thalidomide plus prednisone regimen (thalidomide 100 mg/day and prednisone 12.5 mg/day). No prophylactic antithrombotic therapy was initiated. After two months of thalidomide plus prednisone therapy, he was admitted to our hospital with acute right arm ischemia, which was emergently treated with thrombectomy of the right brachio-cephalic trunk. On postoperative day one the patient experienced left side weakness with right internal carotid thrombosis documented at duplex scanning and angiography study (Figures 1(a)  and 1(b)). The CT scan at 48 hours after neurological symptoms, showed two acute cerebral infarctions in the frontal lobe with no haemorrhage. No cardiovascular risk or procoagulant risk factors were found, and the basic coagulation parameters were normal before surgery. Other embolic sources were excluded by ECG and transthoracic echocardiography. 

 Thalidomide treatment were the only risk factor associated with arterial thrombosis and therefore was immediately stopped. Enoxiparine (4000 IU twice a day) and acetil-salicilic acid (ASA-100 mg/die), was administrated for five days, with subsequent administration of warfarin (INR range 2–2.5) and ASA 100 mg/day. 

 With anticoagulation therapy the patient was free of further signs of new ischemic events. 

 After discharge was initiated a rehabilitative program, with complete regression of arm and leg weakness in 1 month.

## 3. Discussion

Thalidomide is one of the most used drugs in the treatment of newly diagnosed and relapsed/refractory MM and is currently used together with cytotoxic chemotherapy because of its antiangiogenic properties. Continuos low dose of thalidomide and low dose prednisone was part of maintenance program posttransplant [1–3]. The thalidomide's antiangiogenic mechanism is thought to involve blocking (vascular endothelial growth factor) VEGF and fibroblast growth factor activity, resulting in increased MM cell apoptosis [2]. Thalidomide is likely to determine a prothrombotic effect due to a decrease in anticoagulant proteins and an increase in platelet aggregation and procoagulants proteins; however this prothrombotic mechanism is not completely known yet [4]. The prothrombotic effect can be increased by additional cytotoxic or corticosteroid therapy, due to endothelial damage or expression of procoagulant factors [4, 5]. 

 While the increased thrombo-embolic risk of thalidomide therapy is widely described in the venous system [6, 11, 12], the arterial complications are scarcely known. A review of the literature using the keywords “Thalidomide AND arterial thrombosis AND multiple myeloma—limits: English” allowed us to find out 11 previous cases [6–10], as shown in Table 1. In literature [6–10] is described five cases of cerebral thrombosis associated with stroke; one case complicated with mortality [8] and six cases of limb ischemia, one case complicated with major amputation [8]. None of these cases was under anticoagulation or antiplatted therapy at symptoms onset. All patients experienced an unique ischemic event in a precise anatomic region, no recurrent symptoms were found. 

 Our case is peculiar because of two consecutive arterial thrombosis events in two different anatomic region. Probably the association of both thalidomide and corticosteroid increases the thrombotic risk due to synergic effect of the two drugs. 

 The highest risk of thrombotic events with the thalidomide use is within the first months of induction therapy, particulary when thalidomide is concomitantly used with high dose cytotoxic agents. A prophylactic antithrombotic therapy by antiplatelet and anticoagulant drugs is mandatory in these cases, however no studies are available to confirm this assumption [13, 14].

## 4. Conclusion

Thalidomide is one of the most widely used drugs on the treatment of MM because of its antiangiogenic properties. Because of the rare but severe arterial complication, this side effect should be expected when the thalidomide therapy is administered, especially with concomitant cytotoxic chemotherapy or corticosteriod therapy. Until future studies on the mechanism of thalidomide are available, prophylactic therapy with ASA or anticoagulation during thalidomide administration is mandatory.

## Figures and Tables

**Table 1:** Arterial complication during Thalidomide administration for MM.

Author	N	Localization	Cytotoxic/corticosteroid therapy	TEE* prophylaxis
Bowcock 2002 [6]	2	Cerebral	None	none
Scarpace 2005 [7]	4	Tibial-Cerebral	Cytotoxic and corticosteroid VAD°protocol	none
Ortin 2006 [8]	1	Cerebral	Cytotoxic and corticosteroid	none
Goz 2007 [9]	2	Ilio-femoral-popliteal	VAD°protocol	none
Altinas 2007 [10]	2	Femoral-Tibial	VAD°protocol and corticosteroid	none

*TEE (thrombo-embolic events). °VAD protocol (vincristine, adriamycine, dexamethasone).

## References

[1] D'Amato R, Loughnan M, Flynn E, Folkman J (1994). Thalidomide is an inhibitor of angiogenesis. *Proceedings of the National Academy of Sciences of the United States of America*.

[2] Knight R (2005). IMiDs: a novel class of immunomodulators. *Seminars in Oncology*.

[3] Barlogie B, Tricot G, Anaissie E (2006). Thalidomide and hematopoietic-cell transplantation for multiple myeloma. *New England Journal of Medicine*.

[4] Van Heeckeren WJ, Sanborn SL, Narayan A (2007). Complications from vascular disrupting agents and angiogenesis inhibitors: aberrant control of hemostasis and thrombosis. *Current Opinion in Hematology*.

[5] Zonder JA (2006). Thrombotic complications of myeloma therapy. *Hematology*.

[6] Bowcock SJ, Rassam SM, Ward SM, Turner JT, Laffan M (2002). Thromboembolism in patients on thalidomide for myeloma. *Hematology*.

[7] Scarpace S, Hahn T, Roy H (2005). Arterial thrombosis in four patients treated with thalidomide. *Leukemia and Lymphoma*.

[8] Ortin X, Rodriguez-Luaces M, Calabuig M, Font L (2006). Stroke in a multiple myeloma patient treated with thalidomide. *Journal of Stroke and Cerebrovascular Diseases*.

[9] Goz M, Eren MN, Cakir O (2008). Arterial thrombosis and thalidomide. *Journal of Thrombosis and Thrombolysis*.

[10] Altintas A, Avvildiz O, Atay AE, Cil T, Isikdogan A, Muftuoglu E (2007). Thalidomide-associated arterial thrombosis: two case reports. *Annals of the Academy of Medicine Singapore*.

[11] Zangari M, Anaissie E, Barlogie B (2001). Increased risk of deep-vein thrombosis in patients with multiple myeloma receiving thalidomide and chemotherapy. *Blood*.

[12] Urbauer E, Kaufmann H, Nösslinger T, Raderer M, Drach J (2002). Thromboembolic events during treatment with thalidomide. *Blood*.

[13] Baz R, Li L, Kottke-Marchant K (2005). The role of aspirin in the prevention of thrombotic complication of thalidomide and anthracycline-based chemotherapy for multiple myloma. *Mayo Clinic Proceedings*.

[14] Zangari M, Barlogie B, Anaissie E (2004). Deep vein thrombosis in patients with multiple myeloma treated with thalidomide and chemotherapy: effects of prophylactic and therapeutic anticoagulation. *British Journal of Haematology*.

